# Iodide-mediated Cu catalyst restructuring during CO_2_ electroreduction†

**DOI:** 10.1039/d1ta11089f

**Published:** 2022-05-03

**Authors:** Aram Yoon, Jeffrey Poon, Philipp Grosse, See Wee Chee, Beatriz Roldan Cuenya

**Affiliations:** Department of Interface Science, Fritz Haber Institute of the Max Planck Society Berlin 14195 Germany swchee@fhi-berlin.mpg.de roldan@fhi-berlin.mpg.de

## Abstract

Catalyst restructuring during electrochemical reactions is a critical but poorly understood process that determines the underlying structure–property relationships during catalysis. In the electrocatalytic reduction of CO_2_ (CO_2_RR), it is known that Cu, the most favorable catalyst for hydrocarbon generation, is highly susceptible to restructuring in the presence of halides. Iodide ions, in particular, greatly improved the catalyst performance of Cu foils, although a detailed understanding of the morphological evolution induced by iodide remains lacking. It is also unclear if a similar enhancement transfers to catalyst particles. Here, we first demonstrate that iodide pre-treatment improves the selectivity of hexagonally ordered Cu-island arrays towards ethylene and oxygenate products. Then, the morphological changes in these arrays caused by iodide treatment and during CO_2_RR are visualized using electrochemical transmission electron microscopy. Our observations reveal that the Cu islands evolve into tetrahedral CuI, which then become 3-dimensional chains of copper nanoparticles under CO_2_RR conditions. Furthermore, CuI and Cu_2_O particles re-precipitated when the samples are returned to open circuit potential, implying that iodide and Cu^+^ species are present within these chains. This work provides detailed insight into the role of iodide, and its impact on the prevailing morphologies that exist during CO_2_RR.

## Abstract 2

“Changes in electrocatalyst morphology induced by iodide pre-treatment and during CO_2_ reduction are visualized using electrochemical cell TEM, revealing how adding iodide leads to stabilized Cu^+^ and I^−^ species, and selectivity towards C_2+_ products”.

## Graphical abstract

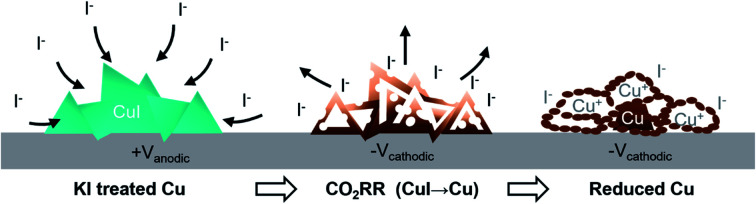

## Introduction

Electrocatalytic carbon dioxide reduction (CO_2_RR) and water splitting, powered by clean and renewable energy resources, are widely explored reactions due to their promise as sustainable approaches to generate fundamental feedstock molecules, such as H_2_, CO and hydrocarbons.^1^ However, most of these technologies still require better catalysts to support the electrochemical reactions.^2^ In particular, there is a lack of in-depth understanding of a catalyst's dynamic structure under reaction conditions, which is however critically needed to inform rational catalyst design. There is also increasing evidence that significant catalyst restructuring can already occur as soon as the catalysts are placed into an electrolyte and these changes impact the subsequent catalyst activity and selectivity during reaction.^3^ For example, it is known that the electrocatalytic performance of Cu, the most favourable catalyst material for generating hydrocarbons in CO_2_RR,^4^ can be altered by using electrolytes that contain different ion species.^5–9^ Interestingly, restructured copper surfaces, such as those derived from Cu oxide/hydroxide,^10–13^ anodic pulsing^14,15^ or those created *via* halide pre-treatments,^16–23^ also commonly produce more energy dense products such as ethylene and oxygenates (C_2+_ products) than pristine well-ordered metallic copper.^24^ A common observation is that these restructured Cu surfaces are often characterized by significant roughness, although the details differ depending on the treatment.^12,13,18,22,23,25,26^ Despite the increasing utilisation of electrolyte-induced restructuring as an electrode preparation method, there have been few attempts to look at the morphological transformations in detail and explore avenues for the controlled synthesis of the most beneficial nanostructures for a given electrocatalytic process.

The presence of halides in the electrolyte is known to create highly restructured Cu surfaces with varying structure depending on the halide species and different catalytic properties, and with iodide producing the most significant improvement in selectivity for CO_2_RR.^19–22,25^ So far, the transformation of Cu in different halides has only been tracked with either space-averaged *in situ* techniques, such as X-ray diffraction, X-ray absorption spectroscopy (XAS)^22,26^ and Raman spectroscopy,^25^ which lack the precise spatial resolution to capture detailed morphological information, or *ex situ*/quasi-*in situ* methods, where the preservation of the original Cu chemical state is uncertain. For example, studies using bulk-sensitive *operando* XAS and surface-sensitive quasi-*in situ* X-ray photoelectron spectroscopy (XPS) have revealed the existence of Cu^+^ (ref. 19 and 20) and surface adsorbed halides during and after reaction.^20,23^ However, the intricate relationship combining both morphological and chemical aspects makes it difficult to deconvolute the contributing parameters towards higher C_2+_ product selectivity without microscopic insight into the prevailing structures that exist under reaction conditions. Furthermore, these earlier works focused on the pre-treatment of single-crystal and polycrystalline Cu foils,^20–22,25^ or heavily oxidised Cu foils.^19,20^ How these transformations translate to the dispersed and particulate catalysts that are more relevant to real world electrocatalysis applications remains unclear.

In this work, we describe the effect of an iodide pre-treatment on the morphology of Cu islands and the subsequent evolution of these islands during and after CO_2_RR visualized using *ex situ* and *in situ* electron microscopy. First, we designed Cu islands arranged in ordered hexagonal arrays as model pre-catalysts and confirmed that these samples replicated both, the morphological changes and the enhancement in C_2+_ selectivity due iodide pre-treatment as previously reported in Cu foils.^19–22^ Then, using *in situ* electrochemical liquid cell transmission electron microscopy (EC-TEM), we captured the formation of CuI tetrahedra from these islands during iodide pre-treatment and their subsequent abrupt transformation into porous, filament-like structures during CO_2_RR in iodide-free electrolyte. Furthermore, the removal of the applied potential led to unexpected re-structuring, where Cu_2_O and CuI particles grew within the filaments. These *in situ* observations, starting from well-defined pre-catalysts, allow us to elucidate the role of residual iodide in controlling the catalyst working morphology in iodide-free electrolyte and in stabilizing Cu^+^ species *via* CuI formation and decomposition. Our work also provides insights into the possibility of tuning the working morphology of electrocatalysts and how the presence of iodine can serve to boost C–C coupling processes during CO_2_RR.

## Results and discussion

### Synthesis of ordered arrays of Cu islands

**Fig. 1:**
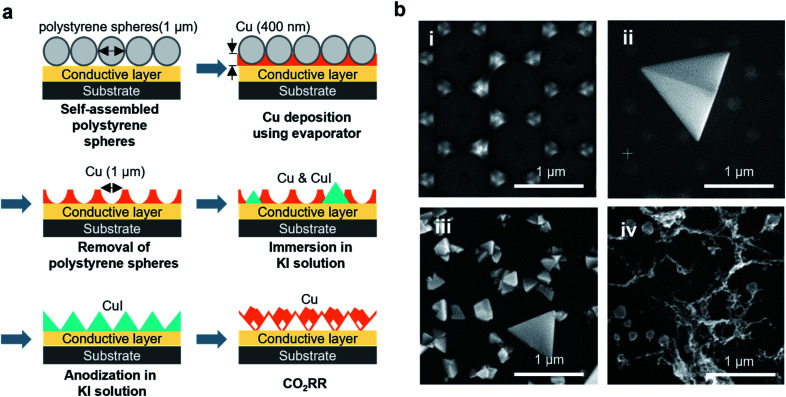
Synthesis of Cu island arrays used to monitor Cu restructuring. (a) Cartoon showing the preparation of a Cu electrode for CO_2_RR: 1 μm-large polystyrene spheres were self-assembled and monodispersed on the desired support. Then, a 400 nm-thick Cu layer was deposited using an e-beam evaporator. The Cu islands are exposed after removal of the polystyrene layer. The Cu array transforms into CuI after immersion and anodization in a KI aqueous solution. The CuI electrode then restructures into porous Cu filaments under CO_2_RR conditions. (b) SEM images of the Cu island structure after (i) the removal of the polystyrene spheres, (ii) immersion in 0.1 M KI solution, (iii) anodization in 0.1 M KI solution and (iv) CO_2_RR in iodide-free CO_2_ saturated 0.1 M KHCO_3_.

Fig. 1 describes the preparation of the hexagonal Cu island array using nanosphere lithography. The lateral size and interparticle distance of the Cu islands were controlled by laying down a mask of 1 μm diameter polystyrene nanospheres on the support surface. Subsequently, 400 nm of Cu was deposited using physical vapour deposition. The removal of the polystyrene spheres afterwards leaves behind a hexagonal array of Cu islands that are ∼350 nm wide (Fig. 1b(i)) with the interparticle distance defined by the sphere diameter (see the Experimental section for details). These Cu islands were deposited on the bulk substrates, such as glassy carbon plates or Au-coated silicon wafers for the *ex situ* selectivity measurement or on the carbon electrode of a microfabricated EC-TEM chip. In particular, these ordered Cu islands define the reaction area more clearly than a Cu film or foil, thereby providing unique opportunities for *in situ* microscopy studies of reaction-induced restructuring. It was reported previously that KI treatment of Cu foils led to the formation of CuI tetrahedra.^21,22^ While we found large micrometer-sized tetrahedra scattered among the Cu islands after iodization in 0.1 M KI (Fig. 1b(ii)), such re-structuring did not occur uniformly over all the synthesized Cu islands, with many nearly pristine Cu islands still present on the substrate (ESI Fig. 1†). We also highlight here that the micrometer size CuI tetrahedra are about 3–4 times larger than the initial Cu islands, which implies significant dissolution and mass transport of Cu in the presence of iodide ions. To generate the CuI tetrahedra more uniformly, we added an anodization step to promote the pre-oxidation of Cu, which facilitated CuI formation, as suggested by previous works.^20–22^ Indeed, CuI tetrahedra were then found uniformly over the entire sample area after anodization, as shown in the scanning electron microscopy (SEM) images provided in Fig. 1b(iii), showing the role of surface oxidation in determining the iodide treated morphology. The resultant morphology of the Cu catalysts after CO_2_RR is shown in Fig. 1b(iv).

### Impact of restructuring on the catalytic performance of CO_2_RR

**Fig. 2:**
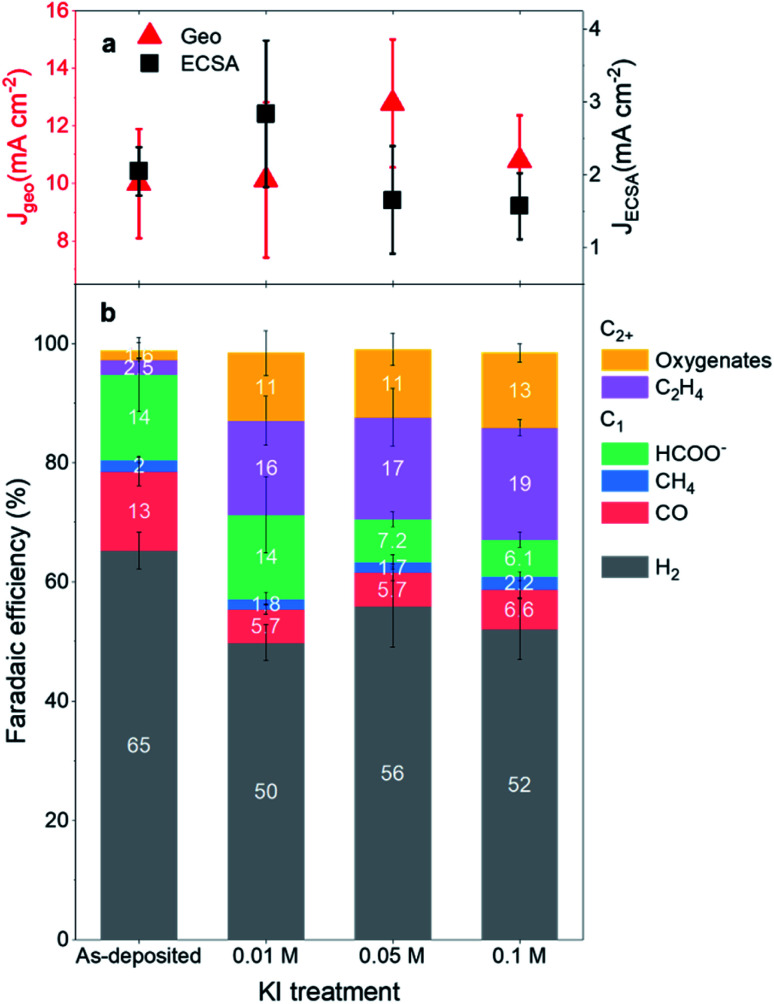
Changes in the CO_2_RR activity and product selectivity as a function of the KI pre-treatment. (a) Geometrical current density and ECSA-normalized current density of the non-treated and KI pre-treated copper arrays. (b) FE of H_2_ and CO_2_RR products including CO, CH_4_, C_2_H_4_, HCOO^−^ and oxygenates. The reaction was conducted in iodide-free and CO_2_-saturated 0.1 M KHCO_3_ at −1.0 V_RHE_ for 1 hour. The error bar is the standard deviation of the repeated measurements on three different samples that were prepared with identical procedures.

**Fig. 3:**
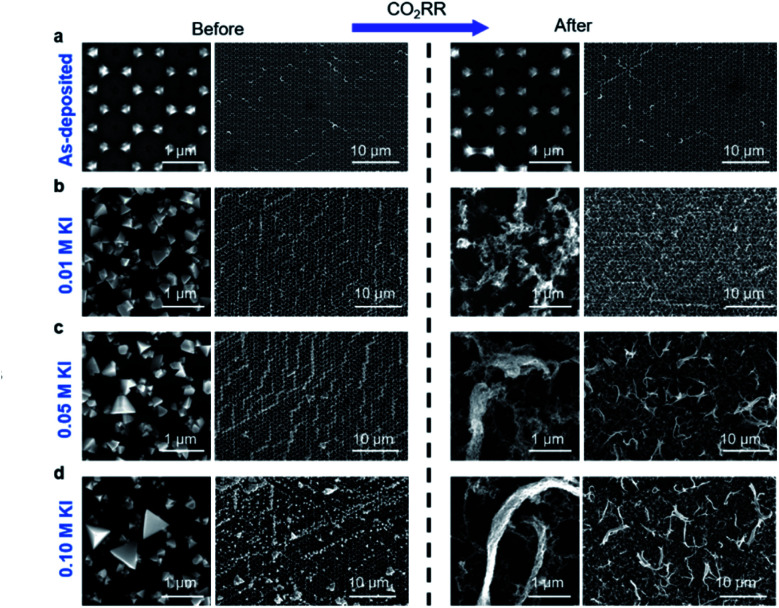
Copper restructuring before and after CO_2_RR. The Cu-island arrays were pre-treated with different concentrations of KI solutions and then subjected to CO_2_RR in iodide-free CO_2_ saturated 0.1 M KHCO_3_ electrolyte at −1.0 V_RHE_ for 1 hour. SEM images taken after pre-treatment are shown on the left, and the same samples after CO_2_RR are shown on the right: (a) control (no KI exposure), (b) 0.01 M KI, (c) 0.05 M KI, and (d) 0.1 M KI. The samples were prepared on Au-coated silicon wafers.

To understand the impact of such restructuring on the catalytic performance of the Cu islands, we prepared the Cu arrays in KI solutions of different concentrations and then reacted them in iodide-free CO_2_-saturated 0.1 M KHCO_3_. Fig. 2 and 3 show the changes in catalytic activity, selectivity and morphology of these Cu pre-catalysts as a function of the KI pre-treatment concentration measured at −1.0 V_RHE_ in an iodide-free CO_2_-saturated 0.1 M KHCO_3_ solution. The current densities normalized by the sample geometry as well as by the electrochemical surface area (ECSA) are shown in Fig. 2a, while the calculated faradaic efficiencies (FE) of the CO_2_RR products of these samples are displayed in Fig. 2b. The methods for the calculation of the ECSA and FEs are outlined in the ESI Table 1 and Notes.†

The product analysis indicates a clear improvement in the catalytic selectivity for C_2+_ products with pre-treatment in KI concentrations of 0.01 M and above, but the activity trend as a function of the KI concentration is less clear. The geometrical current density initially increased at 0.05 M KI, but subsequently slightly decreased for the 0.1 M KI. Conversely, the ECSA-normalized current density was found to decrease for KI concentrations ≥0.05 M. Nonetheless, no matter the normalization method used, the changes observed are small, and we consider that such fluctuations in the current densities are not likely the result of a real intrinsic change in the activity of our Cu catalysts. Instead, they may be explained by a competition between the increases in surface area caused by the restructuring process being offset by the loss of Cu material due to dissolution at higher iodide concentrations, or by mass transfer limitations in the porous structures.

On the other hand, the C_2+_ selectivity improvements over that of untreated Cu islands were similar for the three pre-treatment concentrations. The FE of ethylene (C_2_H_4_) and oxygenates (alcohols, aldehydes, and acetate) increased by 25%, while the FE of hydrogen (H_2_) decreased by about 15%, and that of formate (HCOO^−^) and carbon monoxide (CO) together decreased by more than 7%. No clear selectivity trends are seen for methane (CH_4_) production, but its yield in the untreated Cu samples was also very low.

The H_2_ production is relatively high in these samples compared to similar literature results because of the exposed Au support (used as a conductive layer on the silicon wafer), which at relatively high reductive potentials encourages the hydrogen evolution reaction.^27,28^ While having Au as a conductive layer underneath the copper could influence the overall C_2+_ selectivity, we can still see that there is an enhancement due to the iodide pre-treatment. Au is also not known to produce hydrocarbons and oxygenates at −1.0 V_RHE_ and only produces CO.^4^ Hence, it is reasonable to assume that the carbonaceous products (especially the C_2+_) are generated by the Cu arrays and conclude that C_2+_ (C_2_H_4_ and oxygenates) products are promoted in the iodide pre-treated-Cu samples at the expense of the two-electron CO_2_RR reduction products (formate and carbon monoxide).^28,29^ We also repeated the iodization experiments on Cu arrays deposited on glassy carbon plates and they revealed similar enhancements in C_2+_ product selectivity (see ESI Fig. 2†). We can, therefore, confirm that the beneficial effect of iodide pre-treatments during CO_2_RR transfers to particulate electrocatalysts irrespective of the support employed.

The SEM images shown on the left columns of Fig. 3 further describe the influence that the KI concentration has on the restructuring of the pre-catalysts after iodide pre-treatment, and the corresponding morphologies found after CO_2_RR on the right columns. At a low KI concentration of 0.01 M, we predominantly form small tetrahedra with a fairly uniform size distribution over the entire sample area. With increasing KI concentration, we see the appearance of larger tetrahedra with sizes in the 1000 to 3000 nm range. Here, these changes are explained by the increasing iodide concentration dissolving more Cu oxide and re-distributing Cu away from the initial array, which in turn led to the creation of larger tetrahedra.

These characteristics of the iodide-treated samples also impacted the electrocatalyst morphology found after CO_2_RR as compared to the untreated samples. As seen from Fig. 3b–d, even a pre-treatment with a low iodide concentration led to a drastic restructuring during electrolysis, turning the CuI tetrahedra into long filament-like structures after reaction, whereas the untreated Cu islands remained similar to their as-synthesized morphology (Fig. 3a). These filaments also appeared similar to the after-reaction structures found on iodide treated Cu foils reported previously.^20^ In this case, due to the well-defined nature of our Cu array pre-catalysts, we can unambiguously associate these filaments to the CuI tetrahedra. As shown in Fig. 3c and d, larger filaments are formed with increasing KI concentrations.

### *In situ* observation of the electrocatalyst transformations during CO_2_RR

To confirm that these filaments also existed under reaction conditions and to better understand the restructuring induced by iodide, we studied the Cu-island arrays in parallel using *in situ* and *ex situ* TEM. The Cu arrays were prepared on a carbon electrode of EC-TEM chip with the same method we used for Cu arrays on Au-coated silicon wafers, and then we traced the morphology and the chemical changes that occurred in Cu arrays during anodization/iodization and the subsequent CO_2_RR.

**Fig. 4:**
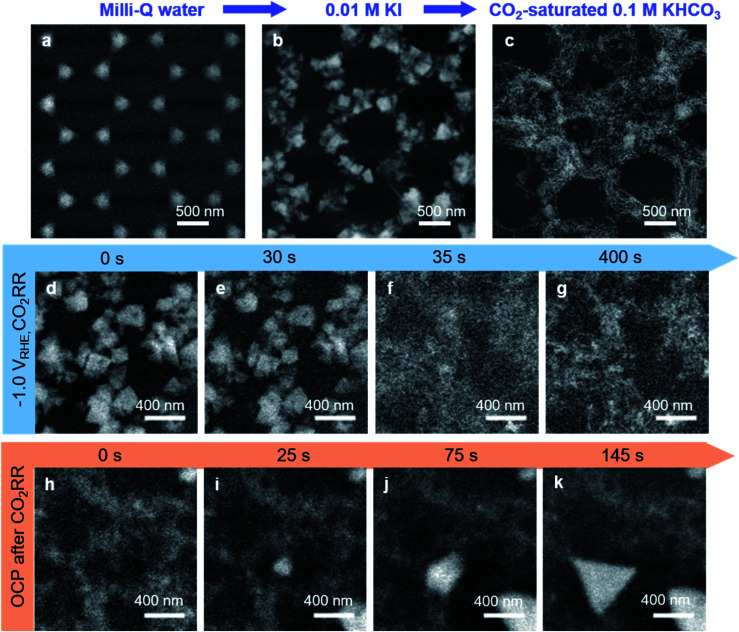
Structural transformation of lithographically-deposited Cu islands monitored *via in situ* EC-TEM in STEM mode using a high angle annular dark field detector. STEM images of the Cu arrays immersed in different electrolytes are shown: (a) in ultrapure water, (b) 0.01 M KI after anodization, and in (c) iodide free-CO_2_ saturated 0.1 M KHCO_3_ after 30 minutes of CO_2_RR at −1.0 V_RHE_. (d–g) CuI transformation into Cu filaments under CO_2_RR conditions (−1.0 V_RHE_, iodide free-CO_2_ saturated-0.1 M KHCO_3_). (h–k) After 30 minutes of CO_2_RR, the potential was removed, and triangular CuI structures re-precipitated at OCP over time. The images are averaged by 5 frames to enhance the signal-to-noise ratio (d–k).

Fig. 4a–c display representative scanning transmission electron microscopy (STEM) images of the Cu electrode morphology as-prepared, after anodization in an aqueous KI solution and the resultant structures after CO_2_RR. As shown in Fig. 4a, the Cu islands were stable in Milli-Q water at open circuit potential (OCP, 0.15 V_RHE_). Next, we exchanged the electrolyte by flowing a 0.01 M KI aqueous solution for 10 minutes without applied potential. With the electrolyte exchange, the Cu islands spontaneously reduced in size, and small tetrahedral particles appeared on at the periphery of the Cu islands (ESI Fig. 3 and Movie 1†). Then, to promote complete CuI tetrahedra formation, two anodic cyclic voltammetry (CV) treatments between OCP and 0.60 V_RHE_ at 10 mV s^−1^ were applied. The pre-catalyst morphology consisting of CuI tetrahedra after anodization is shown in Fig. 4b. The associated CV is shown in ESI Fig. 4.† There, a singular oxidative peak can be seen, indicating Cu^0^ oxidation to Cu^+^, dissolving Cu and driving the formation of the stable and poorly soluble CuI particles instead of CuO formation.

After anodization in 0.01 M KI, the electrolyte was replaced with iodide-free CO_2_-saturated 0.1 M KHCO_3_ at a constant flow rate of 1.25 ml min^−1^, and a negative potential was applied to observe the catalyst evolution under CO_2_RR (Fig. 4d–g). CO_2_RR was performed against the same Ag/AgCl reference electrode where the applied potential was chosen such that we had a converted potential of −1.0 V_RHE_. In KHCO_3_, the CuI tetrahedra slowly decomposed over time (ESI Fig. 5†), but under an applied negative potential, the decomposing tetrahedra experienced drastic structural and chemical transformations. In the initial linear sweep voltammetry (LSV) when the applied potential was scanned from OCP to −1.0 V_RHE_ at 15 mV s^−1^, a reduction peak appeared in the curve prior to CO_2_RR, as seen in ESI Fig. 6,† lending evidence to the transformative reduction from Cu^+^ to Cu^0^.^30^ After about 30 seconds at −1.0 V_RHE_, the CuI particles lost their tetrahedral shape and transformed into a network of filaments.

**Fig. 5:**
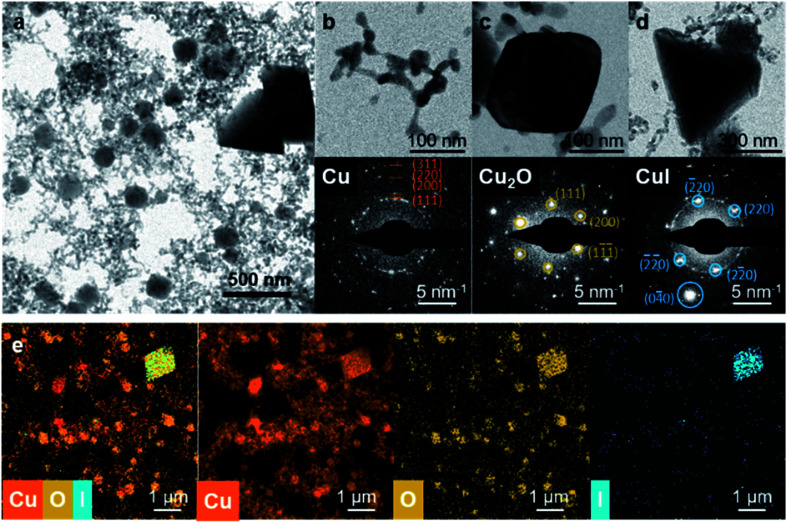
Morphology and crystallographic structure of iodide pretreated-Cu after CO_2_RR. (a) TEM image of the after-reaction structures showing Cu filaments, sporadic Cu_2_O octahedra, and CuI tetrahedra. (b) Cu, (c) Cu_2_O, and (d) CuI structures are shown with their respective selected area electron diffraction patterns. (e) STEM-EDX images of filament-like structures after CO_2_RR, featuring three distinctive elements, Cu, O, and I.

Fig. 4e and f and ESI Movie 2† show the transformation of tetrahedral CuI into Cu filaments during chronoamperometry at −1.0 V_RHE_. In the short time between *t* = 30 s to *t* = 35 s during chronoamperometry (Fig. 4e and f), the CuI particles abruptly transformed into faint thread-like structures. These structures further consolidated into more distinct filaments as shown by the appearance of the smaller nanometer-sized bright spots (Fig. 4f and g), but no further large-scale changes were seen over the entire duration of the electrolysis until the cathodic potential was removed. The reconstructions after removing the applied potential were especially interesting. We saw that the samples spontaneously re-oxidized at OCP and tetrahedral structures reappeared within the filament network, seen as a bright contrast amid the weaker background contrast in Fig. 4h–k and ESI Movie 3.†*Ex situ* electron diffraction and STEM-energy dispersive X-ray spectroscopy (EDX) characterization (shown later in Fig. 5) confirmed that the tetrahedral structures were CuI, indicating the presence of residual iodide in the filamentous structures since there was no iodide in the KHCO_3_ electrolyte. In a separate experiment (ESI Fig. 7 and Movie 4†), we show that the CuI particles can further dissolve and reform during additional cycling from OCP to cathodic conditions and back to OCP, suggesting that the residual iodide species are surface-adsorbed.

### Further analysis of the Cu structure and re-oxidation after CO_2_RR

We also characterized the *in situ* TEM sample after reaction in more detail with *ex situ* TEM. Here, we can see that there were, in fact, three distinctive structures in the absence of applied potential; the Cu filaments, Cu_2_O and CuI based on electron diffraction and EDX as shown in Fig. 5. The different structures are easily discernable by their shape and size (Fig. 5a–d) as well as their respective different oxygen and iodine contents as determined through EDX mapping (Fig. 5e). Cu oxide (Cu_2_O) has an octahedral shape with a size between 100–200 nm (Fig. 5c). CuI particles have tetrahedral or octahedral shape with larger sizes, from 500 nm to a micrometer (Fig. 5d), but with a much lower number density than the tetrahedral structures seen in the samples after anodization/iodization in Fig. 3. The re-precipitated CuI and Cu_2_O particles were also found in the Cu arrays deposited on bulk glassy carbon supports after CO_2_RR (ESI Fig. 8†), confirming that they were not artifacts of the EC-TEM experiment.

More importantly, images of the filaments showed that they were made of interlinked chains of small and irregularly shaped Cu nanoparticles (NPs) with an average size of 20 nm (Fig. 5b). A high resolution TEM image of one of such interlinked Cu NPs is presented in ESI Fig. 9.† Both, EDX and selected area electron diffraction also confirmed that the NPs were metallic Cu, in agreement with the reductive peak in the LSV that appeared prior to applying the constant potential (−1.0 V_RHE_) for CO_2_RR.

When the *in situ* and *ex situ* TEM data are considered together, the results suggest that two forms of Cu were present under reaction conditions: Cu^0^ that constitutes the bulk of the filaments and Cu^+^ that is stabilized under reaction conditions but reforms into the re-precipitated particles after the removal of the applied potential. We emphasize here that the Cu_2_O and CuI particles cannot be explained by the re-oxidation of metallic Cu because if that is the case, we should only find oxidized Cu after reaction. Therefore, the Cu contributing to re-precipitated particles must be a metastable form of Cu. Moreover, since we only found Cu_2_O and CuI particles, the valence state of this metastable ‘Cu species’ is likely related to Cu^+^, which is surprising given the cathodic conditions of CO_2_RR. Nonetheless, the presence of Cu^+^ in samples treated with iodide had been postulated in previous works.^11,19,20,23^ The key role of Cu^+^ in improving the selectivity towards C_2+_ products has also been postulated in other high-performance Cu catalysts for CO_2_RR.^11,15,18–20,31–35^ We also mention here that while we had previously reported similar re-structuring and fragmentation in Cu_2_O cubes during reaction,^3^ the re-precipitation of Cu_2_O particles when the potential was returned to OCP was not seen in those experiments (see ESI Fig. 10† for a comparison with oxidized Cu islands). Hence, these metastable Cu^+^ species were created as a result of the iodide pre-treatment.

Furthermore, the discovery of CuI particles only in selected regions of the samples implies that the amount of iodide left in the samples after reaction was significantly less than the amount that was introduced during pre-treatment. Wide-area STEM-EDX mapping of the iodide-treated sample before and after CO_2_RR (ESI Fig. 11†) confirmed that the iodide content in the filament structures decreased during CO_2_RR. We further surveyed the chemical composition of Cu–I–O in the Cu arrays prepared on the Au coated silicon wafer with SEM–EDX. The chemical composition measured by SEM–EDX over an 8 × 6 μm^2^ area is outlined in ESI Fig. 12 and 13,† plotted with respect to an increasing KI concentration. KI-treated samples suffer a 25–30% atomic percentage fall of the iodine content, and any residual iodide was below the EDX detection limit after CO_2_RR. The inability to pick up a signal of residual iodide with SEM-EDX is, however, not surprising because recent XPS work had suggested that the amount of iodide remaining on the Cu surface after CO_2_RR can be as low as 0.1 to 0.36 at%.^23^

**Fig. 6:**
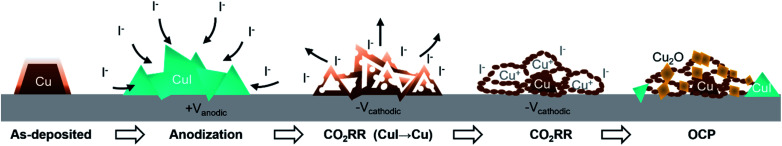
Schematic describing the proposed evolution of the iodine-pretreated Cu islands after I-anodization and under CO_2_RR condition. The morphological, volumetric, and chemical changes are featured. The different chemical states of Cu are represented by the different colours, metallic Cu in brown, Cu_2_O in dark yellow, and CuI in cyan.

In Fig. 6, we illustrate how the morphology of iodide-treated Cu electrocatalysts evolves during iodine anodization as well as during CO_2_RR and provide a plausible explanation of the drastic changes observed. An as-deposited Cu island transforms to CuI during anodization in the KI-containing solution. Under CO_2_RR conditions, CuI is reduced back to Cu. We hypothesize that during the application of a cathodic potential, the significant volumetric difference between CuI and metallic Cu causes the formation of dispersed and fragmented Cu NPs. The filaments, in turn, result from the self-assembly of these NPs. The fragmented nature of these NPs likely also contributes to the stabilization of the Cu^+^ and I^−^ species, which then appear as the re-precipitated Cu_2_O and CuI particles when the potential is returned to OCP.

In current literature, there are three prevailing hypotheses regarding the role of iodide in improving the selectivity of Cu; the first being an effect of iodide adsorbed on the surface of Cu or embedded in electrical double layer,^17^ the second the stabilization of Cu^+^ by iodide,^19,20,23^ and the third a roughness increase by restructuring.^22^ While our results showed that the KI pre-treatment induced extensive re-distribution of Cu on the electrode surface, the restructuring was more than a simple roughening of the electrode surface. The CuI tetrahedra were volumetrically much larger than the initial Cu islands in both, non-anodized samples (Fig. 1b(ii)) and anodized (Fig. 1b(iii) and 3d–f), indicating that there was significant reordering of Cu due to the presence of iodide ions. The CuI tetrahedra themselves undergo complete reorganization once a reductive potential is applied in iodide-free CO_2_-saturated 0.1 M KHCO_3_, forming long filaments made of interlinked and irregularly shaped Cu NP aggregates. Despite such extensive changes in catalyst morphology, it is unlikely that the selectivity improvement was a result of increased roughness since the selectivity did not change significantly with pre-treatments at different KI concentrations even though the treatments led to markedly different surface structures. Our observations showing the re-precipitation of CuI and Cu_2_O particles within the Cu filaments upon returning to OCP, on the other hand, supported that stabilized Cu^+^ and iodide species were present under reaction conditions. In addition, the Cu_2_O NPs re-precipitated at a higher frequency compared to CuI particles, implying that Cu^+^ is the more likely contributor to the selectivity improvement.

The localized nature of CuI re-precipitation meant that iodide could be still found in significant amounts within certain regions of the Cu filaments, which would explain why iodide was detected in previous work using ensemble averaging measurements such as XAS and XPS. Hence, we hypothesize that the formation of CuI from the oxidized Cu by pre-treatment and its subsequent transformation in 3-dimensional filaments under CO_2_RR conditions gives rise to a morphology that is favourable for the dynamic stabilization of Cu^+^/Cu interfaces which are beneficial for the formation of C_2+_ products. We do not, however, fully rule out the effect of surface-adsorbed iodide or that iodide plays a role in stabilizing the Cu^+^ species. Although there can be some differences between our dispersed Cu islands and a bulk Cu foil that can supply nearly infinite amounts of Cu towards CuI formation, the mechanism responsible for the selectivity enhancement should be similar in both cases, given that the filaments were also seen to form on the surface of foil samples. More importantly, these results suggest that we should be able to improve C_2+_ selectivity by optimizing the restructured Cu network morphology through tuning the pre-treatment parameters.

## Conclusions

We presented a detailed picture of how Cu islands restructure under the influence of I^−^ ions before, during, and after CO_2_RR. We observed a unique morphology made out of filamentous structures formed by the transformation of iodide-treated copper, CuI, to Cu^0^ and Cu^+^ at cathodic potential during CO_2_RR. Despite a significant loss of iodide during reaction, we observed the re-precipitation of tetrahedral CuI and Cu_2_O particles when returning to OCP. The reappearance of the copper iodide particles is the direct evidence that residual iodide species remain on the Cu filament surfaces. These observations also indicate that Cu^+^ species are stabilized in the filamentous structures under the cathodic conditions of CO_2_RR. The latter might explain the beneficial effect of iodide pre-treatments for steering the selectivity of Cu particle catalyst towards hydrocarbons and oxygenates, since Cu/Cu^+^ interfaces have been suggested to favour C–C coupling. Furthermore, this work demonstrates how *in situ* electron microscopy in conjunction with templated catalyst deposition can be used to gain mechanistic insight into the dynamic re-structuring of catalysts under reaction conditions, which affects their performance and is broadly applicable to other electrocatalytic reactions.

## Experimental methods

### Cu electrode preparation

Flat Cu electrodes were prepared by electron beam deposition of 100 nm Cu onto gold-plated Si(100) wafers with a 5 nm Ti layer and 10 nm gold overlayer deposited *via* electron beam evaporation.

### Templated Cu electrode preparation

Templated Cu metal spacing was achieved through nanosphere lithography, following a synthetic route detailed in the literature.^36^ In summary, a 300 μL monodisperse 909 nm polystyrene sphere suspension (microParticles GmbH, Berlin, Germany, 909 ± 27 nm, 5 wt% suspension) was added to 300 μL 1% vol^−1^ styrene solution in ethanol and 10 μL of 0.1% vol^−1^ sulphuric acid. This mixture was gently dosed onto the surface of a Petri dish of ultrapure water (18.2 MΩ cm resistivity) through a curved glass pipette for the particles to self-assemble into a hexagonal close packed layer, with large (several centimeter lengths) domain formations and clear iridescence. The layer was consolidated by dosing a dilute surfactant and the electrode substrates were placed underneath the assembled layer into the water. The water was gently drained using a siphoning tube, leaving the assembled layer onto the substrate for drying. Position-controlled Cu electrodes were prepared by physical vapor deposition of 400 nm Cu onto the substrates using an e-beam evaporator. Gold-plated silicon wafers (Au(10 nm)/Ti(5 nm)/Si(100)) were used as electrode substrates. The polystyrene spheres were subsequently removed by 30 s sonication in ultrapure water and rinsed with ultrapure water. The substrate was then blown dry with compressed air.

### Cu electrode preparation using anodization

Iodide treated-Cu was prepared using anodization in KI solution. The templated Cu electrodes were immersed in the KI solutions (0.01, 0.05, and 0.1 mM) for 10 minutes for conditioning. The anodic potential was applied from 0 to 0.45 V (where the first adsorption peak appeared) against the OCP with a scanning rate of 10 mV s^−1^.

### Electroreduction of CO_2_ and product analysis

Electrochemical experiments were conducted using a H-type cell, separating the cathodic and anodic compartments with a Selemion AMV ion exchange membrane (AGE Engineering Co., Ltd, Chiba, Japan). A platinum mesh (99.95%, Advent Research Materials Ltd, Oxford, UK) counter electrode, reversible hydrogen electrode (RHE, Mini HydroFlex, Gaskatel, Kassel, Germany) reference, and a modified glassy carbon working electrode were used. The glassy carbon working electrode was held using a polyether ether ketone (PEEK) sample holder. As electrolyte, 0.1 M KHCO_3_ (≥99% purity, Fisher Chemicals) was purified with regenerated Chelex 100 (50 g l^−1^, Bio-Rad Laboratories, Berkeley, USA) to remove trace metal impurities.^37^ Prior to electrochemical measurements, the electrolyte was saturated with CO_2_ (99.95%, Air Liquide Germany, Düsseldorf, Germany) by bubbling the gas 30 min prior to the experiment at an average rate of 20 ml min^−1^, and bubbling was continued throughout the experiment. The pH of the electrolyte at carbon dioxide saturation was 6.8. The experiment was controlled by a Multi Autolab M204 potentiostat (Metrohm-Autolab, Filderstadt, Germany). Throughout the experiments, the working electrode compartment was continuously stirred to ensure that its surface was exposed to a gas-saturated electrolyte and to prevent bubbles from blocking the reference electrode. Each electrolysis was measured for one hour under chronoamperometry mode. The actual potential applied, *V*_applied_, was corrected by: *V*_applied_ = *V*_initial_ − *iR*, with *V*_initial_ the initial applied potential, *R* the sample resistance, and *i* the current at the *V*_initial_, characterized by linear sweep voltammetry from −0.3 V to −1.05 V (*vs.* RHE) prior to chronoamperometric electrolysis. Electrochemical surface area measurements (ECSA) were conducted by using the double layer capacitance method after electrolysis. Cyclic voltammetric scans at a low current potential window between −0.3 V to −0.55 (*vs.* RHE) were used to minimize the possibility of faradaic contributions.

On-line analysis of gaseous products from the cell was done with a gas chromatograph (GC, Agilent 7890B), equipped with a thermal conductivity detector (TCD) and flame ionization detector (FID). Injections occurred every 15 min. Formate, acetate, and 1-propanol were detected by high performance liquid chromatography (HPLC, Shimadzu Prominence, Duisburg, Germany) with a NUCLEOGEL SUGAR 810 column with a refractive index detector (RID). Alcohols were detected through liquid–gas chromatography (Shimadzu 2010 plus) equipped with a fused silica capillary column and a FID. All liquid products were measured after 1 hour of electrolysis, with their FEs calculated. In Fig. 2, FEs are averaged over the repeating measurement on three different samples. A description on the method used for the calculation of the FE of the gas and liquid products can be found in the ESI.†

### *In situ* transmission electron microscopy

The *in situ* EC-TEM experiments were performed in a Thermo Fisher 300 kV Titan TEM (Thermo Fisher Scientific) operated in STEM mode using a Hummingbird Scientific Generation V Bulk Liquid Electrochemistry TEM holder (Hummingbird Scientific) with a Pt counter and Ag/AgCl (3 M KCl) reference electrodes. The image sequences were acquired using an electron probe current of ∼220 pA and at a frame rate of 1 frame per second with 1024 × 1024 pixel image resolution. The electron flux was controlled below 3.5 e^−^ Å^−2^ s^−1^ at all times to minimize electron beam-induced artifacts. The EC-TEM chips with a 50 nm thick silicon nitride membrane window and 250 nm spacer were also produced by Hummingbird Scientific. The EC-TEM chips have a carbon film on the window and act as working electrode. The electrochemistry experiments were performed using a Biologic SP-200 potentiostat. The potentials were measured against the built-in Ag/AgCl reference, calibrated against the Ag/AgCl in a beaker, and then converted to RHE using Nernst's equation. CO_2_RR is performed against the same Ag/AgCl reference electrode.

The TEM holder was pre-filled with Milli-Q water during cell assembly to ensure that the liquid fills the entire fluid path. After loading into the TEM, the syringe was filled with freshly saturated 0.1 M KHCO_3_ and introduced at a flow rate of 1.25 ml min^−1^ for 30 min. Cyclic voltammetry from −0.5 V_RHE_ to −1.3 V_RHE_ was first used to determine the onset potential for the CO_2_RR, followed by chronoamperometry for up to 30 minutes at −1.0 V_RHE_. After chronoamperometry, we stayed at open circuit potential for 10 minutes and continued imaging.

### *Ex situ* scanning electron microscopy and transmission electron microscopy

*Ex situ* microscopy work was conducted using a Thermo Scientific Apreo SEM, with a high stability Schottky field emission gun, a Trinity Detection System,^38^ and an UltraDry energy dispersive X-ray spectroscopy (EDX) detector. The electron imaging and EDX mapping was done with 10 kV acceleration voltage. The EDX was collected for 30 minutes in regions of 8 × 6 μm^2^. For post-mortem TEM analysis, we analysed the same electrochemistry chip used the EC-TEM experiment. After the EC-TEM experiment, the chip was rinsed in Milli-Q water and transferred back into the same electron microscope within 10 minutes. The electron imaging and EDX mapping was done in the same microscope for *in situ* imaging.

## Author contributions

A. Y, J. P., S. W. C., B. R. C, conceived the project. A. Y. and J. P. designed the experiments. A. Y. prepared the samples and performed the electron microscopy work. J. P. developed the lithographical sample preparation method, prepared the samples, and conducted the electrochemical measurements. P. G. assisted with the experiments. A. Y., J. P. and S. W. C wrote the manuscript with the contributions from all authors.

## Conflicts of interest

There are no conflicts to declare.

## Supplementary Material

TA-010-D1TA11089F-s001

TA-010-D1TA11089F-s002

TA-010-D1TA11089F-s003

TA-010-D1TA11089F-s004

TA-010-D1TA11089F-s005
