# Supplementary material for: Iodide-mediated Cu catalyst restructuring during CO2 electroreduction
Source: J Mater Chem A Mater. 2022 May 3;10(26):14041–50. doi: 10.1039/d1ta11089f (PMC9255670; doi:10.1039/d1ta11089f)
Supplement: TA-010-D1TA11089F-s005 [file TA-010-D1TA11089F-s005.pdf]

Electronic Supplementary Material (ESI) for Journal of Materials Chemistry A.
This journal is © The Royal Society of Chemistry 2022

Supplementary Information

Iodide mediated Cu Catalyst Restructuring during CO_2 Electroreduction

Aram Yoon[1ǂ], Jeffrey Poon[1ǂ], Philipp Grosse[1], See Wee Chee[1*], and Beatriz Roldan Cuenya[1*]

[1]Department of Interface Science, Fritz-Haber Institute of the Max Planck Society, Berlin 14195
[ǂ] equal contribution
e-mail: swchee@fhi-berlin.mpg.de; roldan@fhi-berlin.mpg.de

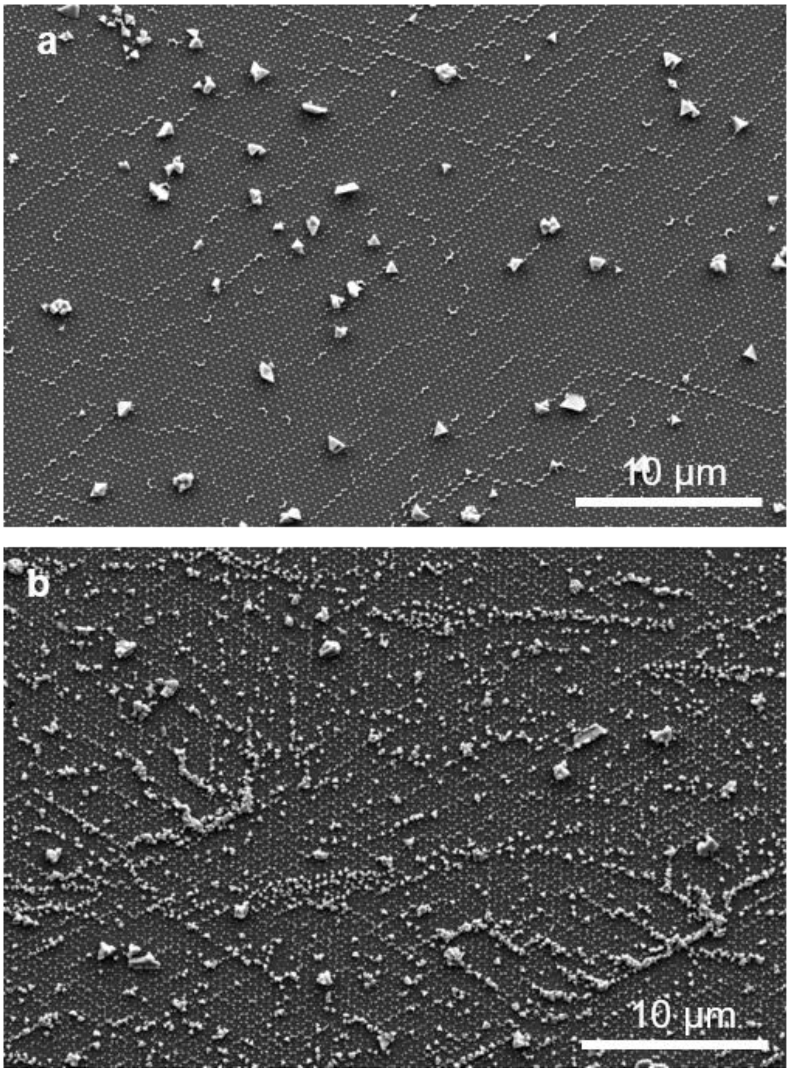

Supplementary Figure 1. Arrays of copper islands restructuring (a) by immersion in a 0.1 M KI aqueous solution for 10 minutes and (b) by anodization (+0.4 V against open circuit potential (OCP))

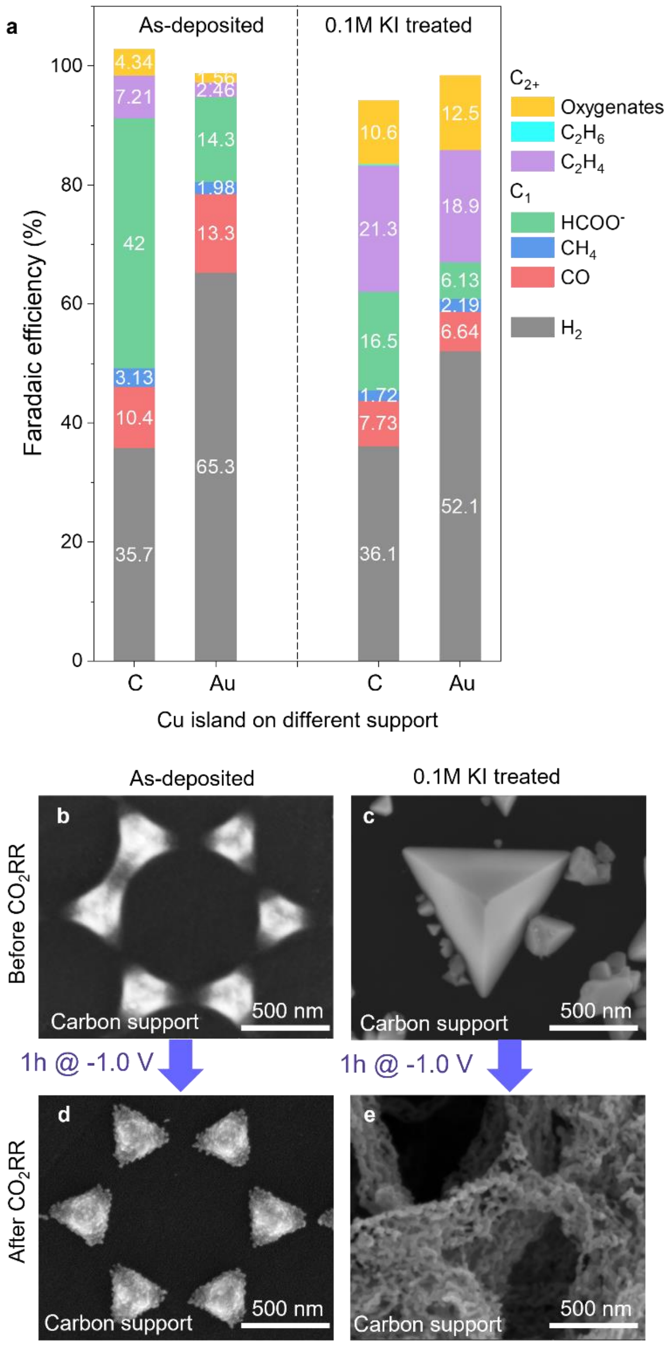

Supplementary Figure 2. Support effect. (a) CO_2RR product selectivity comparison for as-prepared Cu islands vs. 0.1 M KI treated Cu islands on two different supports: glassy carbon (C) and gold (Au). The Au data are the same for the main Figure 2. Faradaic efficiency of H_2 and CO_2RR products including CO, CH_4, C_2H_4, $HCOO^-$ and oxygenates (alcohols, aldehydes, and acetate). The reaction was conducted in iodide-free and CO_2 -saturated 0.1 M $KHCO_3$ at -1.0 V_{RHE} for 1 hour. The increase of C_{2+} selectivity and decrease of HCOO- selectivity are distinct for the KI-treated Cu for both C and Au support. (b-c) SEM images of as deposited (b,d) and 0.1 M KI-treated (c,e) Cu islands on glassy carbon support before (b,c) and after (d,e) CO_2RR.

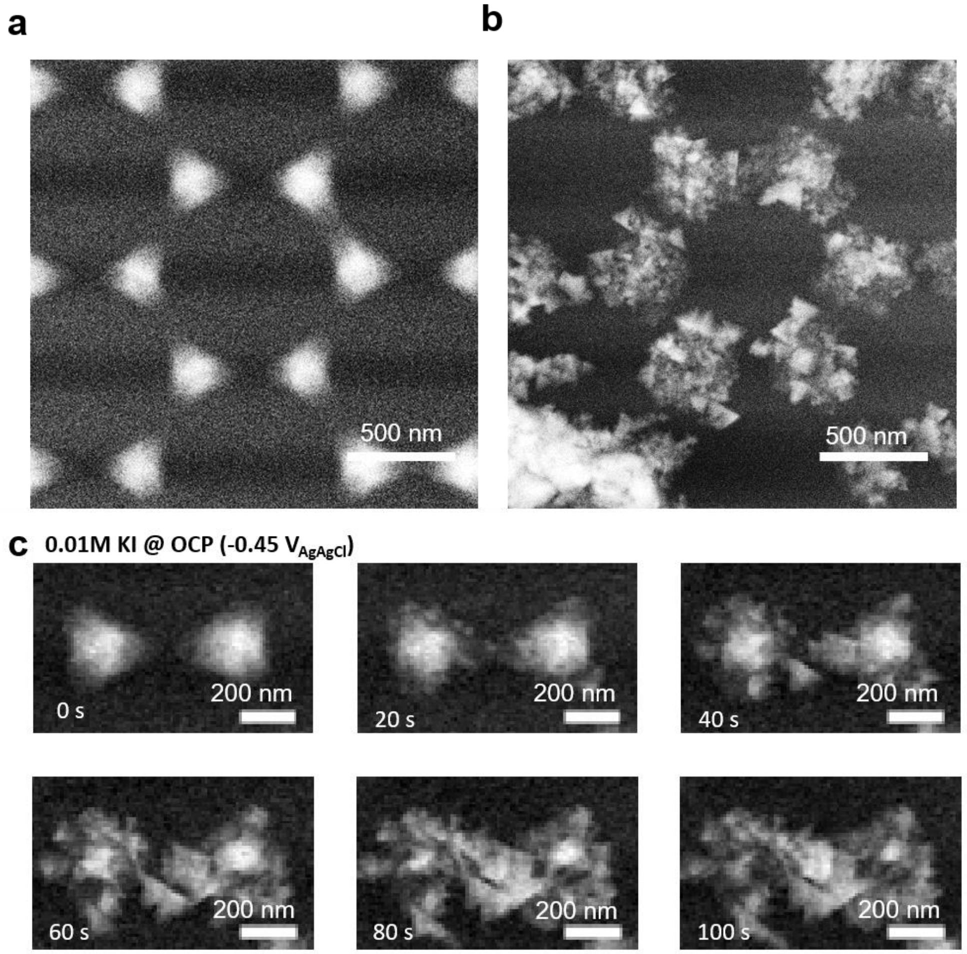

Supplementary Figure 3. Spontaneous iodization by flushing the 0.01M KI into the LC-TEM. (a) the Cu hexagonal array submerged in Milli-Q water. (b) Cu hexagonal array after flushing the 0.01M KI for 10 minutes. (c) Cropped view of the transformation displayed in a time series capturing the dissolution and deposition of triangular CuI structures from the periphery of the Cu islands.

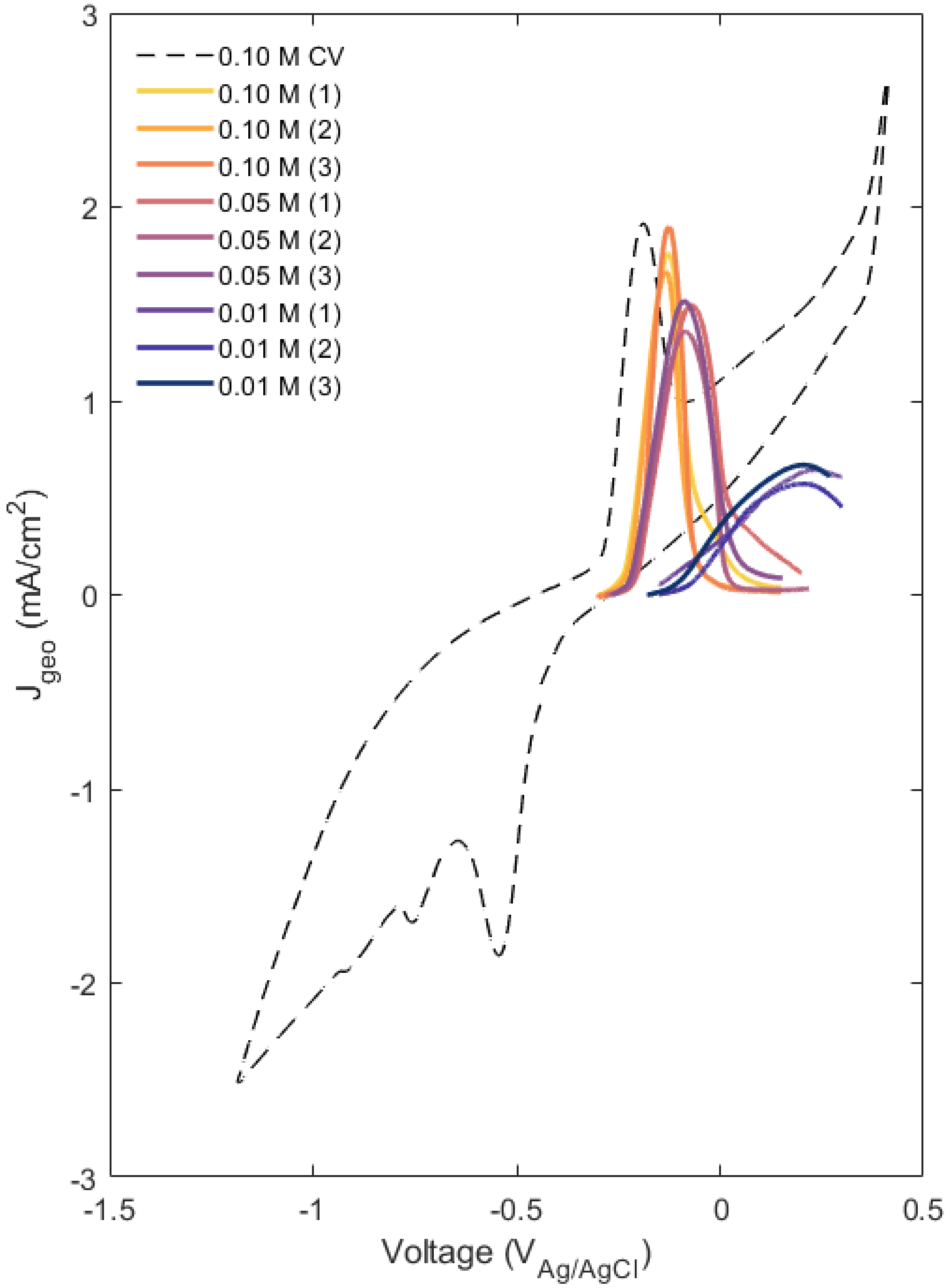

Supplementary Figure 4. Linear sweep voltammetry (LSV) and cyclic voltammetry (CV) curves of copper electrodes in a KI aqueous electrolyte. The CV was obtained from a Cu array by applying the potential clockwise from OCP to -1.2 V_{AgAgCl} at the negative end and to 0.4 V_{AgAgCl} at the positive end. LSV was applied from OCP to +0.4V against OCP with a scanning rate of 10 mV s^{-1}. LSV is repeated three times and the experimental number is denoted in legends.

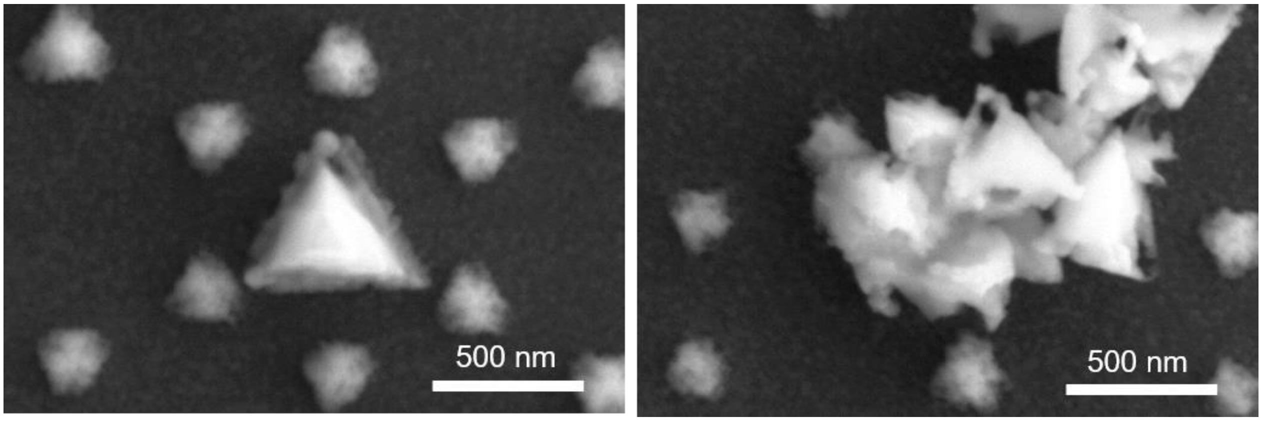

Supplementary Figure 5. Decomposition of the CuI in a CO_2-saturated 0.1M $KHCO_3$ electrolyte by immersion for 10 minutes.

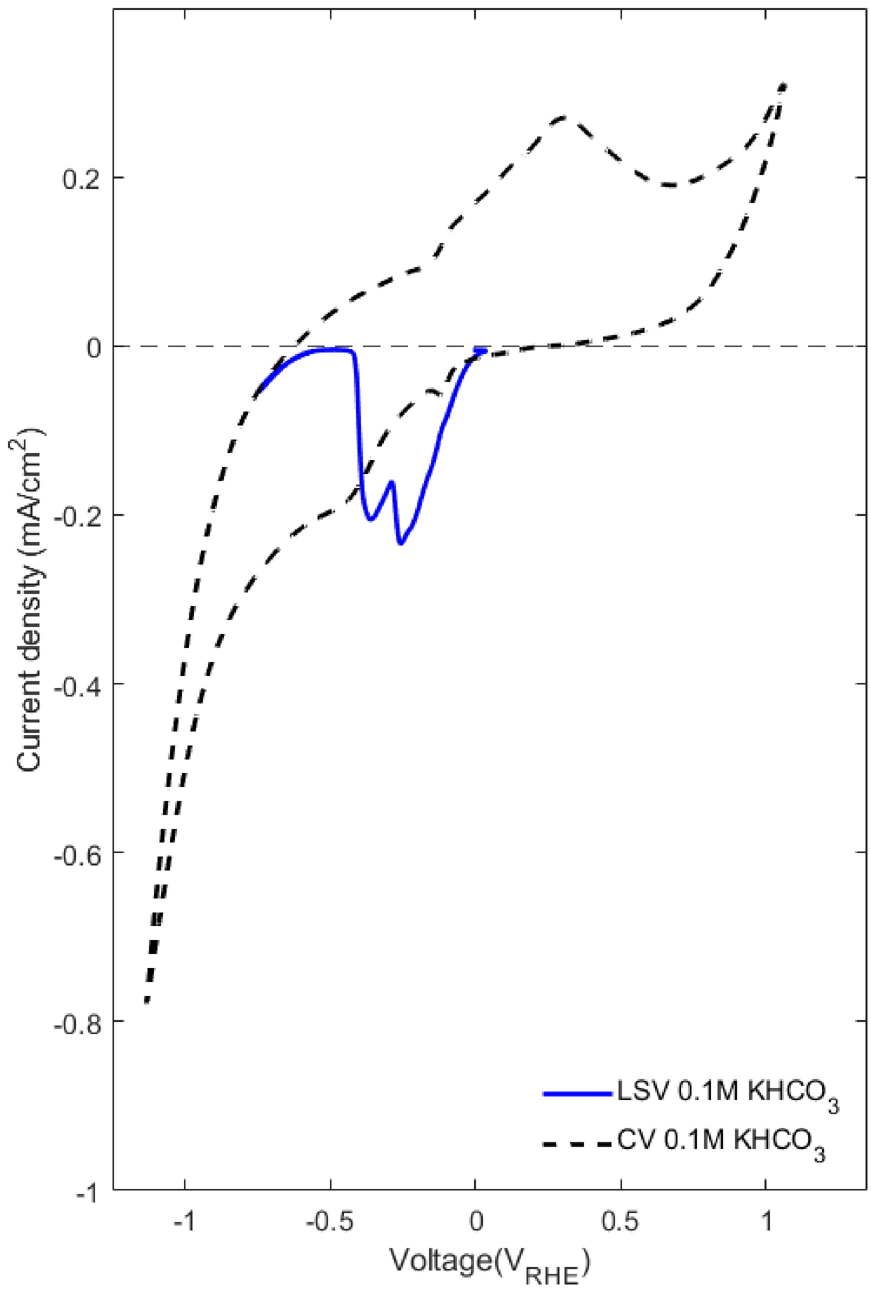

Supplementary Figure 6. CV (dashed) and LSV (solid blue) from 0 V_{RHE} to -1.5 V_{RHE} in CO_2-saturated 0.1M $KHCO_3$. The solid LSV was measured *in situ* (LC-TEM) and the dashed CV is measured from the Cu film in a beaker. The LSV shows a large reductive peak prior to the CO_2RR onset. The LSV potential was taken within the LC-TEM and calibrated with respect to the CV measured in the beaker.

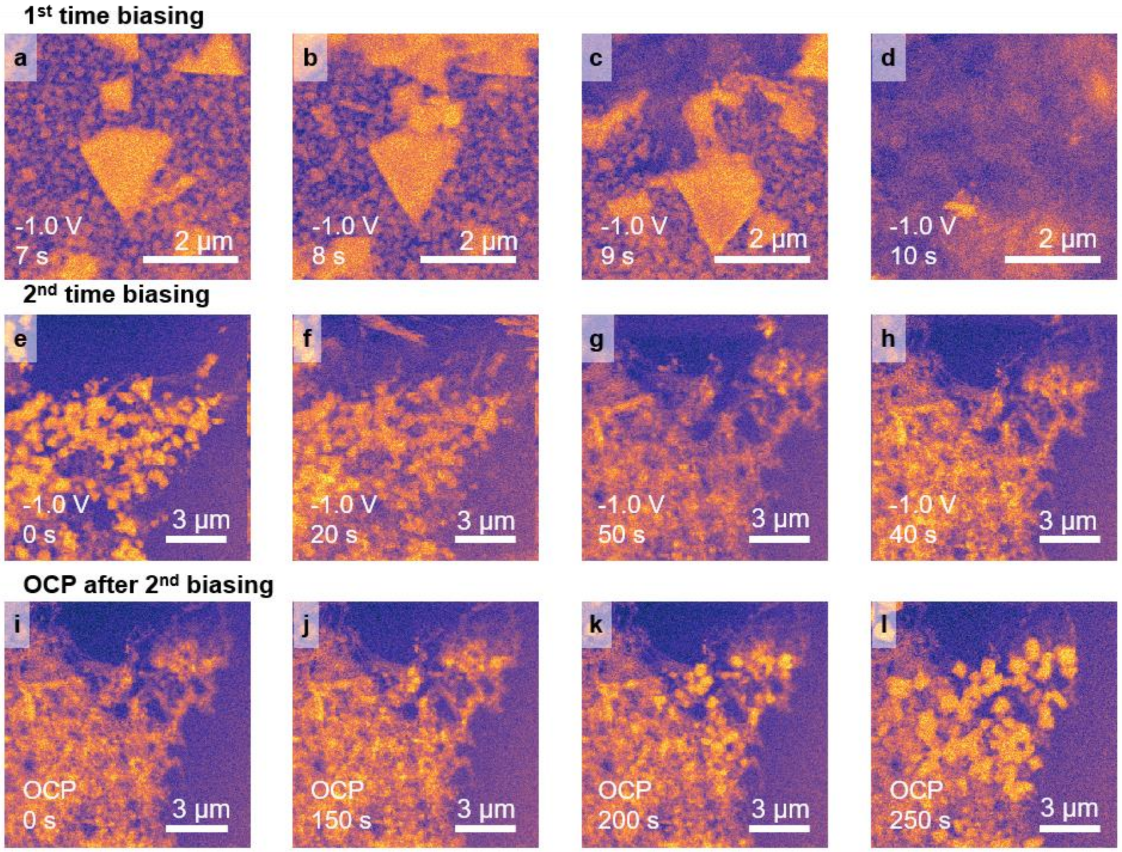

Supplementary Figure 7. Reversible precipitation and disappearance of CuI structures on restructured Cu filaments formed from-iodide treated Cu film observed by LC-TEM during CO_2RR. (a-d) first reduction cycle (e-h) second reduction cycle (i-l) reprecipitation at OCP after removal of the negative potential. All potentials are referenced to the V_{RHE}.

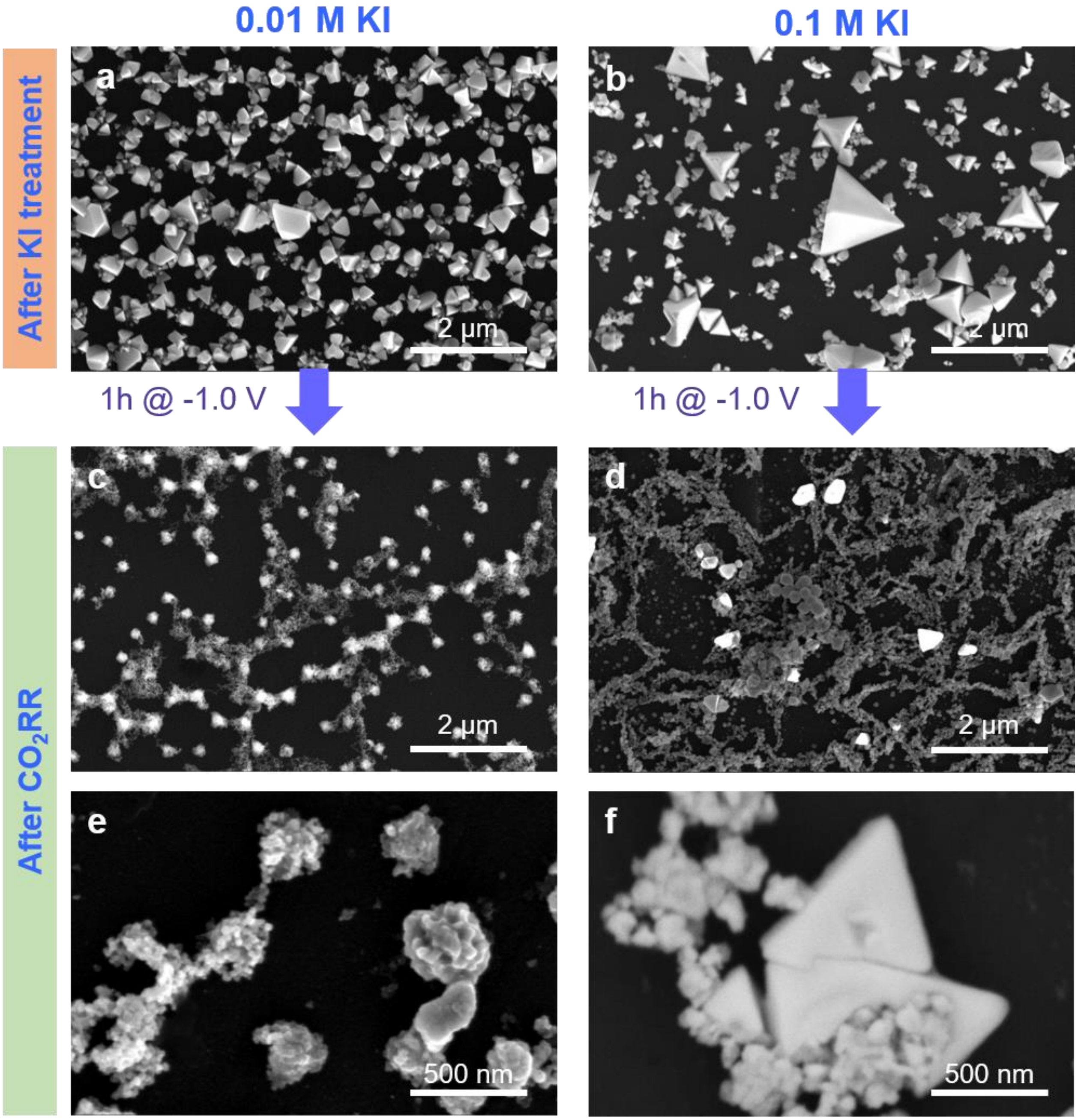

Supplementary Figure 8. SEM imagining of iodide-mediated copper restructuring on glassy carbon before and after CO_2RR. The Cu arrays were anodized in (a) 0.01 M KI and (b) 0.1 M KI. Then, they were transferred to the H-type cell and placed under the CO_2RR conditions (CO_2-saturated 0.1M $KHCO_3$, -1.0 V_{RHE} for 1 hour). (c) and (d) show the respective restructured arrays after CO_2RR. The respective magnified views are shown in (e) and (f). (e) features the remaining template structure and copper filaments. Image (f) shows the tetrahedral CuI formed on top of the Cu filaments.

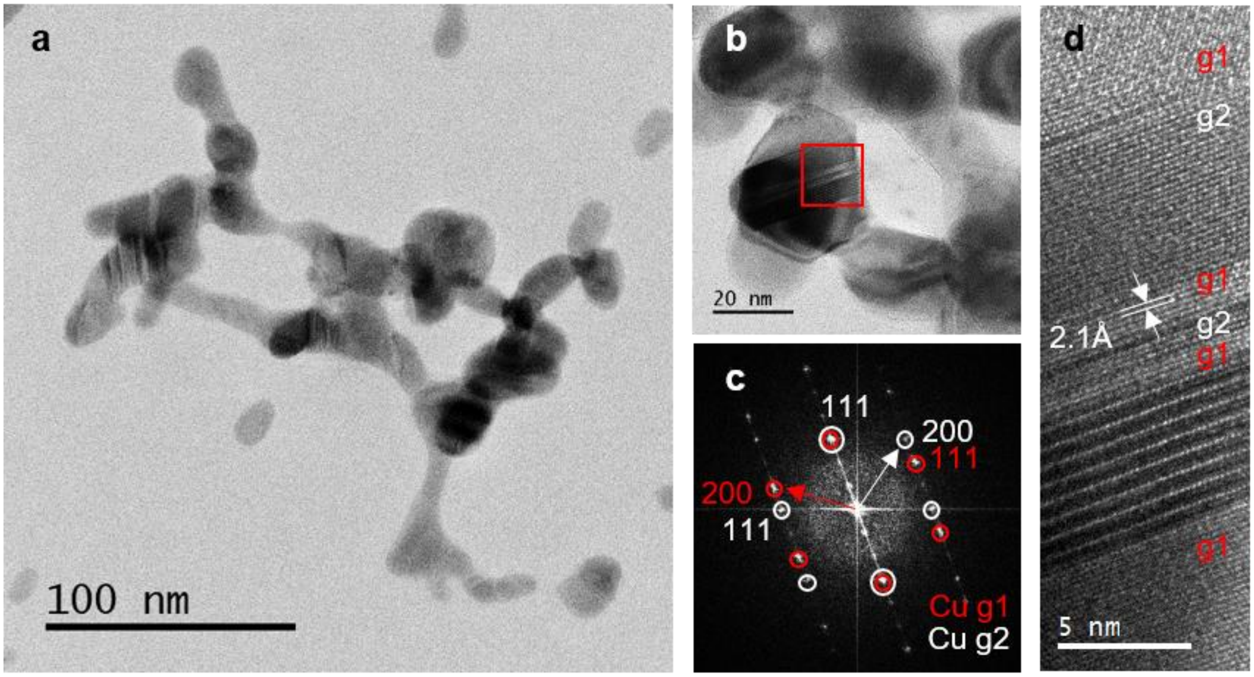

Supplementary Figure 9. TEM images of Cu filaments consisting of small interconnected NPs. (a) A large field of view of Cu filament. (b) A magnified view of a NP. (c) is showing the fast Fourier transform spectrum of the red box area in b. Red marks the peaks from grain 1 (g1) and white marks are from grain2. Two grains have the 109° in-plane rotation while sharing the (1 1 1) plane direction. (d) is showing the high-resolution image of the area highlighted in (b) showing Cu (111) spacing with the contrast variation due to stacking faults.

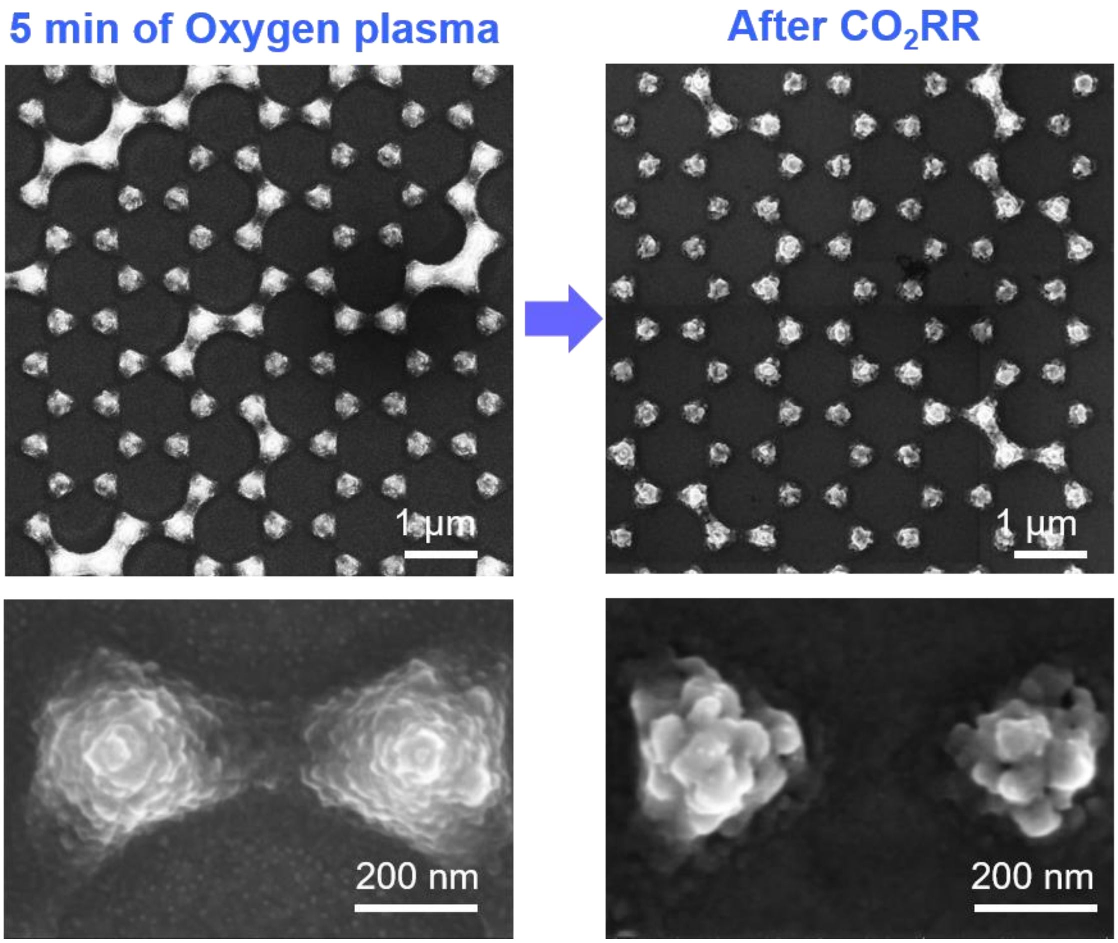

Supplementary Figure 10. Restructuring of oxygen plasma treated-Cu island arrays before and after CO_2RR. For comparison between iodide-treated Cu versus oxygen-treated Cu, we exposed the Cu islands to an oxygen plasma treatment. The oxygen plasma treatment was done for 5 minutes at 20 W at an O_2 pressure of 400 mbar, and induced the oxidation of the Cu islands. After CO_2RR, the arrays of Cu islands are in place almost identical to before CO_2RR, while individual islands restructured, giving rise to the aggregates of the smaller particles.

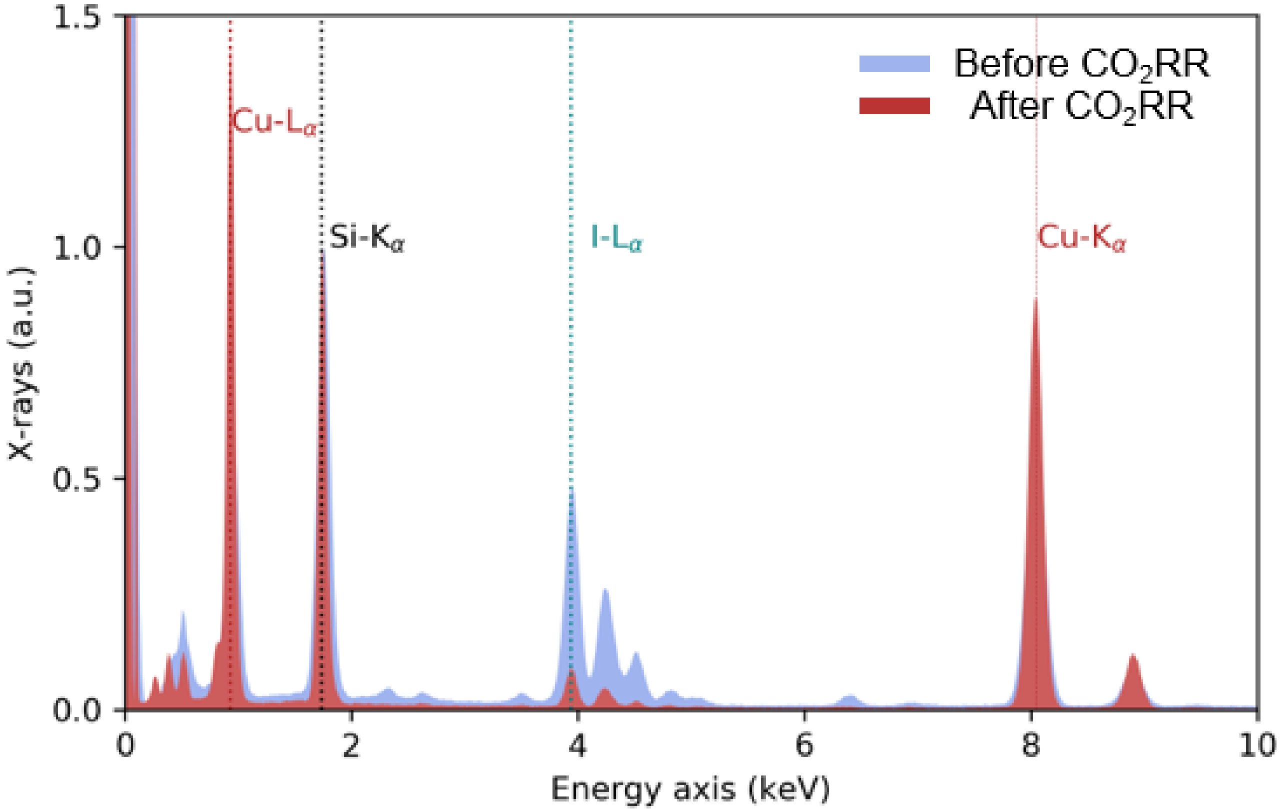

Supplementary Figure 11. Scanning transmission electron microscopy (STEM)-Energy Dispersive X-ray spectrum (EDX) showing the compositional change of the iodide-treated Cu array before (blue) and after (red) CO_2RR. The image was surveyed over 25μm^2 and the x-ray intensity is normalized by the Si-K_α peak intensity which is consistent background from the chip window.

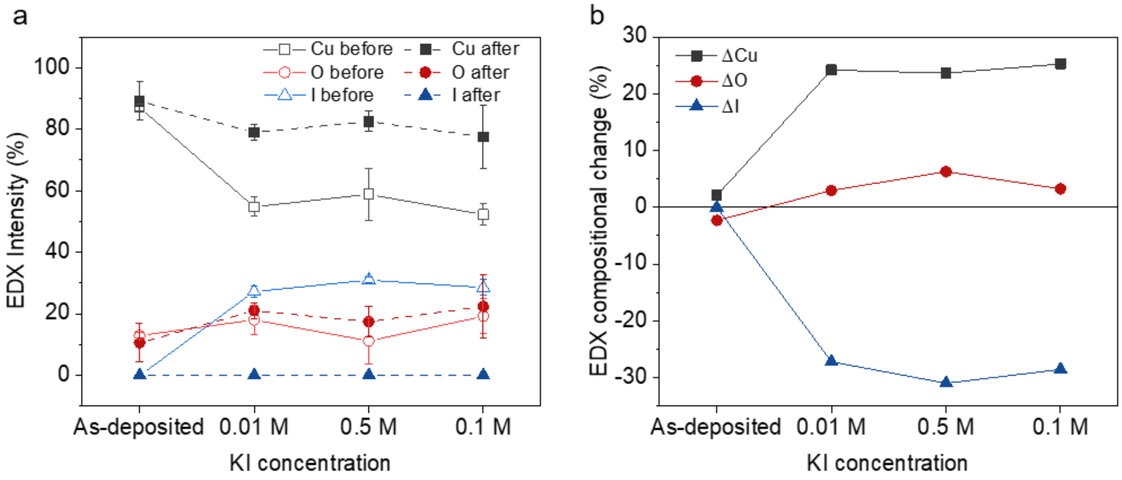

Supplementary Figure 12. SEM-EDX showing the compositional change. (a) relative composition of Cu, I, and O before and after CO_2RR. (b) Difference in the relative composition (ΔCu, ΔO, and ΔI,) defined as atomic percentages after reaction against before reaction, surveyed over an area of 8 × 6 μm^2. We note here that the composition is the relative ratio between Cu, O, and I, but not the absolute amount. The iodide content decreases to an undetectable level after CO_2RR. Prior to CO_2RR, there was a general increase of the surface iodine content up to 30% atomic percentage (blue dash) and saturated above 0.05 M KI, at the expense of Cu atomic (50% atomic percentage, black dash line). Oxygen remains at 20% for all concentrations except 0.1 M KI (red dash) A comparison of the SEM-EDX results before and after iodide treatment are summarized in Supplementary Figure 13.

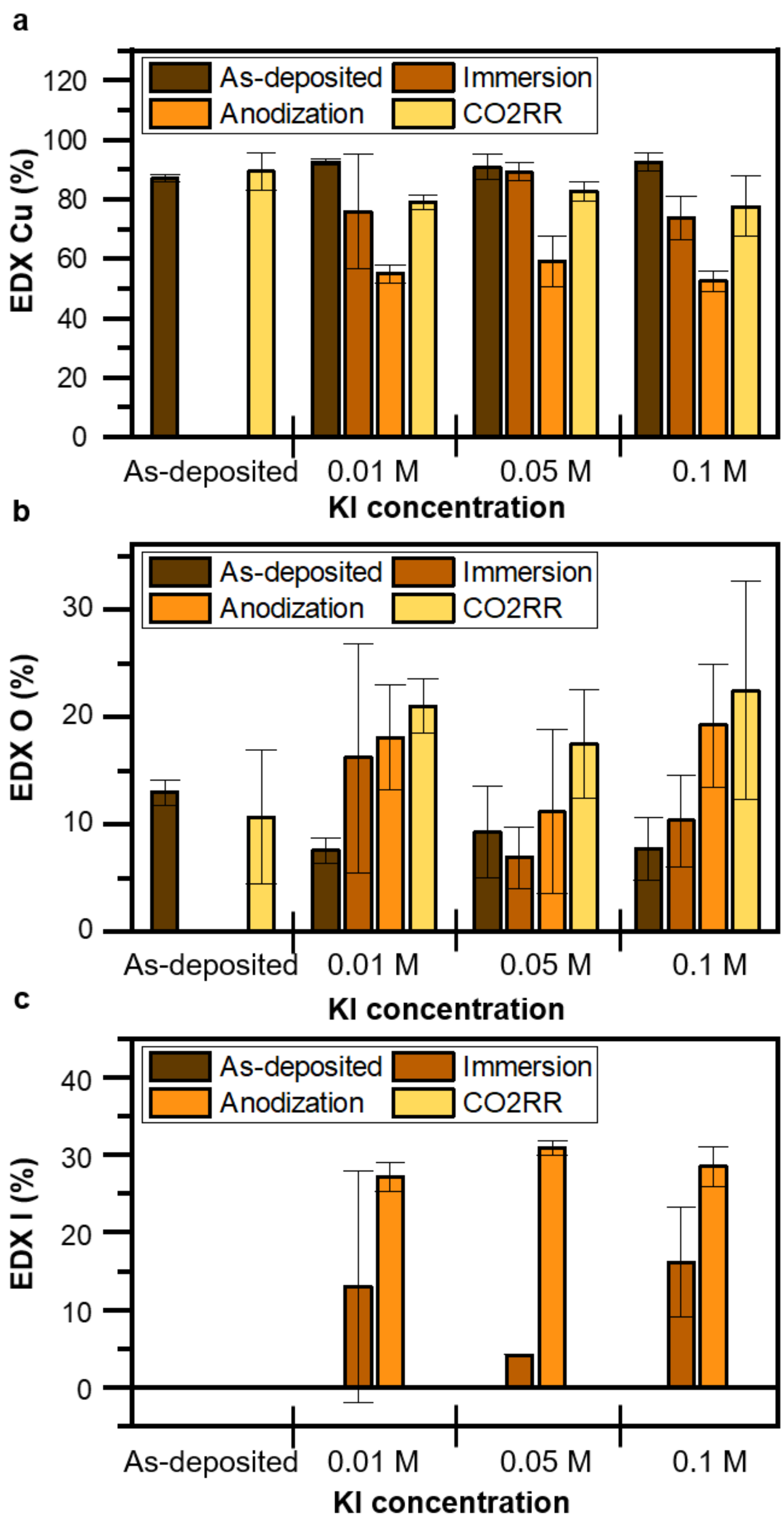

Supplementary Figure 13. SEM-EDX at each stage of the sample preparation: As-deposited, immersion in KI, anodization and CO_2RR. (a) Atomic percent of Cu, (b) Atomic percent of O, and (c) atomic percent of I. The relative composition is calculated by taking Cu + O + I = 100%, so that the composition is the relative ratio between Cu, O and I but not the absolute amount.

Supplementary Note 1.

Electrochemical active surface area of electrode was estimated through a double layer capacitance measurement. The double layer capacitances of each sample were measured via cyclic voltammetry in CO_2 saturated 0.1M $KHCO_3$ solution immediately after CO_2RR. The roughness factor (RF) is calculated by the equation below with the assumption that the only contributing material is copper. The measured capacitance and roughness factors are presented in Supplementary Table 1.

$$RF = \frac{C_{s,i}}{C_{s,Cu}}$$

RF : Roughness factor

$C_{s,i}$: specific capacitance of electrode *i*, μF cm^{-2}

$C_{s,cu}$ *:* specific capacitance of electropolished copper foil, 27 μF cm^{-2}

Supplementary Table 1. Capacitances and roughness factors measured from the Cu island array samples after CO_2RR

Electrode	Capacitance	Roughness Factor
Electropolished Cu foil	27 μF cm^{-2}	1
Cu island as-deposited, after CO_2RR	132 μF cm^{-2}	4.87
Cu island, anodized in 0.01 M KI, after CO_2RR	118 μF cm^{-2}	4.36
Cu island, anodized in 0.05 M KI, after CO_2RR	239 μF cm^{-2}	8.88
Cu island, anodized in 0.10 M KI, after CO_2RR	254 μF cm^{-2}	9.39

Supplementary Note 2. Faradaic efficiency of the CO_2RR gas and liquid products

Calculation of the Faradaic efficiency of gas products:

$$FE_{gas}(\%) = \frac{f_{flow} \times n \times F \times c_{gas}}{I_{total} \times V_m} \times 100$$

FE_{gas}: Faradaic efficiency of gas product, %;

f_{flow}: flow rate of CO_2, mL min^{-1};

I_{total}: total electrolysis current, A;

c_{gas}: volume ratio of gas product, determined by online GC;

V_m: the molar volume of an ideal gas at 1 atmosphere of pressure, 22,400 mL mol^{-1};

n: number of transferred electrons for certain product;

F: Faraday constant, 96,485 C mol^{-1}.

Calculation of the Faradaic efficiency of liquid products:

$$FE_{liquid}(\%) = \frac{c_{liquid} \times V \times n \times F}{Q_{total}} \times 100$$

FE_{liquid}: Faradaic efficiency of a liquid product, %;

c_{liquid}: the concentration of liquid products, determined by HPLC and Liquid GC;

V: the volume of the electrolyte, mL;

n: number of transferred electrons for certain product;

F: Faraday constant, 96,485 C mol^{-1}.

Q_{total}: electrolysis charge, C;

Supplementary Movies

File Name: Movie 1

Description: EC-TEM movie showing the structural transformation of Cu-island arrays into the CuI tetrahedra induced by 0.01 M KI flow at OCP. Five frames were averaged to create one frame of the movie. The movie playback rate is ×25 times real time. The electron flux was 0.43 e^{-} $Å^{-2}$ s^{-1}.

File Name: Movie 2.

Description: EC-TEM movie showing the structural transformation of CuI tetrahedra into filamentous Cu during CO_2RR when the potential was swept from OCP to -1.0 V_{RHE} and the subsequent chronoamperometry (CA) over 37 mins measured at -1.0 V_{RHE} in iodide free -CO_2 saturated-0.1 M $KHCO_3$. The transformation to filaments can be seen around *t* = 3 minutes in the movie, which corresponds to roughly 30 seconds after the start of CA measurements. Five frames were averaged to create one frame of the movie. The movie playback rate is ×100 times real time. The electron flux was 0.43 e^{-} $Å^{-2}$ s^{-1}.

File Name: Movie 3.

Description: EC-TEM movie showing the re-precipitation of Cu_2O octahedra and CuI tetrahedra precipitation at OCP after CO_2RR. Four frames were averaged to create one frame of the movie. The movie playback rate is ×25 times real time. The electron flux was 0.43 e^{-} $Å^{-2}$ s^{-1}.

File Name: Movie 4.

Description: EC-TEM movie showing the effect of potential cycling on an iodide-treated Cu thin film in iodide free-CO_2 saturated-0.1 M $KHCO_3$. The movie starts from the application of -1.0 V_{RHE} to the sample at OCP after 10 mins of CO_2RR. The reprecipitated Cu_2O and CuI structures on copper filaments shortly decomposed under the applied potential but removing potential at around *t* = 18 minutes again led to their re-emergence. Two frames were averaged to create one frame of the movie. The movie playback rate is ×40 times real time. The electron flux was 0.22 e^{-} $Å^{-2}$ s^{-1}.
